# Ongoing Positive Selection Drives the Evolution of SARS-CoV-2 Genomes

**DOI:** 10.1016/j.gpb.2022.05.009

**Published:** 2022-06-26

**Authors:** Yali Hou, Shilei Zhao, Qi Liu, Xiaolong Zhang, Tong Sha, Yankai Su, Wenming Zhao, Yiming Bao, Yongbiao Xue, Hua Chen

**Affiliations:** 1 Beijing Institute of Genomics, Chinese Academy of Sciences and China National Center for Bioinformation, Beijing 100101, China; 2 University of Chinese Academy of Sciences, Beijing 100049, China; 3 Center for Excellence in Animal Evolution and Genetics, Chinese Academy of Sciences, Kunming 650223, China

**Keywords:** COVID-19, SARS-CoV-2, Viral evolution, Natural selection, Darwinian selection

## Abstract

**SARS-CoV-2** is a new RNA virus affecting humans and spreads extensively throughout the world since its first outbreak in December, 2019. Whether the transmissibility and pathogenicity of SARS-CoV-2 in humans after zoonotic transfer are actively evolving, and driven by adaptation to the new host and environments is still under debate. Understanding the evolutionary mechanism underlying epidemiological and pathological characteristics of **COVID-19** is essential for predicting the epidemic trend, and providing guidance for disease control and treatments. Interrogating novel strategies for identifying **natural selection** using within-species polymorphisms and 3,674,076 SARS-CoV-2 genome sequences of 169 countries as of December 30, 2021, we demonstrate with population genetic evidence that during the course of SARS-CoV-2 pandemic in humans, 1) SARS-CoV-2 genomes are overall conserved under purifying selection, especially for the 14 genes related to viral RNA replication, transcription, and assembly; 2) ongoing positive selection is actively driving the evolution of 6 genes (*e.g.*, *S*, *ORF3a*, and *N*) that play critical roles in molecular processes involving pathogen–host interactions, including viral invasion into and egress from host cells, and viral inhibition and evasion of host immune response, possibly leading to high transmissibility and mild symptom in SARS-CoV-2 evolution. According to an established haplotype phylogenetic relationship of 138 viral clusters, a spatial and temporal landscape of 556 critical mutations is constructed based on their divergence among viral haplotype clusters or repeatedly increase in frequency within at least 2 clusters, of which multiple mutations potentially conferring alterations in viral transmissibility, pathogenicity, and virulence of SARS-CoV-2 are highlighted, warranting attention.

## Introduction

A newly emerged betacoronavirus, severe acute respiratory syndrome coronavirus 2 (SARS-CoV-2), causes a worldwide pandemic of the coronavirus disease 2019 (COVID-19), presenting a devastating threat to human public health attributed to its high infectivity and fatality [1], [2], [3], [4]. As of 25 April 2022, COVID-19 has resulted in 509,553,015 worldwide confirmed infections and 6,218,082 deaths across 200 countries and regions (https://coronavirus.jhu.edu/map.html).

RNA viruses usually have high mutation rates and tend to evolve rapidly [5]. For a new RNA virus affecting humans, such as SARS-CoV-2, a recent host shift likely decreases its fitness and impels the virus to adapt to the new host environments and public health interventions [6], [7]. Natural selection may act on the transmissibility and virulence of SARS-CoV-2 through specifically adaptive mutations, which has been observed in Ebola, Zika, and other viruses [6], [7]. SARS-CoV-2 has circulated globally since its first outbreak in December, 2019, and accumulated plenty of genetic mutations within the short period. There are currently more than 5,124,203 complete SARS-CoV-2 genome sequences publicly available, of which 29,735 nucleotide substitutions have been identified (https://bigd.big.ac.cn/ncov/), compiling the largest population genomic data of non-humans so far. The SARS-CoV-2 lineages have exhibited considerable variations in transmission and clinical characters. For example, the basic reproduction numbers (R_0_) range from 2.2 to 5.9 [8], [9], and the mortality rates are from 0.8% to 14.5% [10]. Genetic variants with elevated contagiousness and escape of vaccine-derived immunity emerge and circulate with the outbreaks of Alpha, Delta, and Omicron strains [11], [12].

It is critical for epidemic trend prediction, disease control, and vaccine design to understand whether and how natural selection drives the evolution of transmissibility and virulence of SARS-CoV-2 during the pandemic. If under selection, further research is warranted to identify the functional mutants contributing to the evolving epidemiological and pathogenic characteristics. SARS-CoV-2 evolution is composed of two phases: evolution in animal hosts to obtain the transmission ability to affect human, and in human populations after zoonotic transfer [13]. So far, assessment of natural selection on SARS-Cov-2 is mainly focused on the host shifting phase from animals to humans by analyzing the sequence divergence between SARS-CoV-2 and some closely-related viruses such as BatCoV-RaTG13 [14], [15], [16], [17], [18]. In contrast, few studies target the latter phase due to lack of efficient methods that can handle indeterminate ancestral sequences, extensive sampling bias, and clustering infections of SARS-CoV-2 [13], [14], [19], [20], [21]. Some studies have explored specific mutations that are of potential significance in evolution or molecular function [10], [22], [23], [24]. Nevertheless, the analyses are based on allele frequency change of individual mutations that is not necessarily due to natural selection. It is only useful for screening for the candidate mutant loci instead of serving as a persuasive proof of the presence of natural selection. Despite the individual functional mutants identified in the aforementioned studies, “there is a lack of compelling evidence” of mutations that “impact the progression, severity, or transmission of COVID-19 in an adaptive manner” [6]. Indeed, the evolutionary driving forces underlying the SARS-CoV-2 epidemiological dynamics and pathogenic changes remain elusive. Furthermore, a genome-wide survey of the evolutionary landscape of the functional mutations and their implication of the epidemiological perspective were not fully accomplished either.

Herein, we assess natural selection on SARS-CoV-2 evolution during its pandemic in humans by adopting a novel strategy that is relatively robust to viral clustering infections, founder effects, and sampling bias commonly existing in viral genomic data. The analysis validates the hypothesis that ongoing positive selection is indeed actively acting on the SARS-CoV-2 genomes, shaping the epidemic dynamics of COVID-19. We then partition the viral worldwide samples into clusters according to genomic similarity as a result of global transmission and clustering outbreaks. A spatial and temporal landscape of mutations is constructed on top of the clusters, and the critical mutations potentially conferring pathogenic and clinical characteristics of SARS-CoV-2 are highlighted. Our results provide a reference of viral evolution and genomic mutations for epidemic prediction, surveillance, vaccine design, and clinical treatments of COVID-19.

## Results

A total of 3,674,076 SARS-CoV-2 genome sequences publicly available from 169 countries, as of December 30, 2021, were interrogated, and 69,359 mutations were identified. Since the virus populations are undergoing multiple clustering infections and founder effects, it is hard to use allele frequencies straightforward to draw reliable conclusions on virus evolution [25]. On the premise of neutral evolution, the ratio of nonsynonymous *vs.* synonymous mutations (N_m_/S_m_) should be relatively unaffected by the change in population sizes, since both N_m_ and S_m_ sites from the same gene region are with the common demographic history and should demonstrate identical population behavior when under neutrality. Accordingly in the following sections, our analysis is mainly based on the analysis of N_m_ and S_m_ numbers for different sets of mutations.

### Purifying selection dominates the viral genomic evolution in humans

From diffusion theory for the allele frequency in a large population, the population dynamic of mutations with very low frequency is identical to that of mutations under neutrality, and also, when sample size is very large, the population behavior of deleterious or beneficial mutations when in very low derived frequency is essentially the same to that of neutral mutations [26]. Therefore, we first calculated the N_m_/S_m_ ratio of mutations in very low frequency (*f*) < 0.0001 as 3.17, which represents the relative abundance of N_m_ and S_m_ when the genomes evolve under neutrality. We then checked the relative occurrence of N_m_ and S_m_ in higher frequency. As shown in Table 1, the N_m_/S_m_ ratio of mutations with *f* > 0.001 decreases to 1.28 and is significantly lower than that with *f* < 0.0001 (*Pa* = 2.2E−16, Chi-squared test), indicating a negative selection against N_m_. We further carried out the same analysis for subsets of mutations with different allele frequency ranges (*i.e.*, (0.0001, 0.001] and (0.001, 0.01]). The pattern of reduced N_m_/S_m_ ratios along with increased *f* is consistently observed: the ratio is 1.39 for SNPs with 0.0001 < *f* ≤ 0.001, and is 1.20 for 0.001 < *f* ≤ 0.01. The trend is consistent with the fact that mutations with higher derived allele frequency are usually older than those with lower frequency and are under purifying selection for a longer duration.

**Table 1:** **Chi-squared tests to compare the N_m_/S_m_ ratios between different mutation groups**

**Group**	**Grouping criterion**	**No. of N_m_ sites**	**No. of S_m_ sites**	**N**_m_**/S**_m_**ratio**	**Chi-squaredtest**
Mutations with low frequency of derived alleles	*f* < 0.0001	45,292	14,305	3.17	*Pa* = 2.2E−16 *Pb* = 2.2E−16 *Pc* = 0.0128
Mutations with high frequency of derived alleles (a)	*f* > 0.001	998	778	1.28	
Mutations with high frequency of derived alleles (b)	0.0001 < *f* ≤ 0.001	4407	3181	1.39	
Mutations with high frequency of derived alleles (c)	0.001 < *f* ≤ 0.01	870	722	1.20	
Widespread mutations	> 30 countries	3606	2395	1.51	*P* = 2.2E−16
Non-widespread mutations	< 15 countries	41,398	11,347	3.65	
Mutations with long-time spanning	> 300 days	27,855	14,482	1.92	*P* = 2.2E−16
Mutations with short-time spanning	< 150 days	14,998	1967	7.62	

*Note*: N_m_, nonsynonymous mutation; S_m_, synonymous mutation.

We further compared numbers of N_m_ and S_m_ for widespread (defined as mutations observed in viral samples of more than 30 countries) and non-widespread (defined as mutations observed in samples of less than 15 countries) mutations. The widespread mutations tend to prevail in the populations for a longer time than non-widespread ones. The N_m_/S_m_ ratio of widespread mutations is significantly lower (*P* = 2.2E−16, Chi-squared test; Table 1), suggesting that purifying selection has been acting on these mutations. We also grouped mutations according to their spanning time that was calculated as the duration between the earliest and the most recent collection time of viral genomes carrying the mutations. The N_m_/S_m_ ratio of mutations with long-time spanning (> 300 days) is significantly lower than that of mutations with short-time spanning (< 150 days) (*P* = 2.2E−16, Chi-squared test; Table 1), indicating more selective constraints on long-spanning-time mutations, again, confirming the effect of purifying selection. All the analyses consistently reveal overall purifying selection on SARS-CoV-2 genomes.

### Positive selection drives the adaptive evolution of genes conferring pathogenicity and infectivity

Even though SARS-CoV-2 is under genome-wide negative selection, a small fraction of the viral genome may have undergone positive selection, of which the genetic polymorphism pattern may be diluent in the genome-wide N_m_/S_m_ ratios. To detect positive or purifying selection acting on SARS-CoV-2 individual genes, we again adopted the fact that mutations with higher derived allele frequency usually undergo longer duration of natural selection and present increased/decreased N_m_/S_m_ ratios compared to those with very low frequency, and their population dynamics is identical to those under neutrality. Instead of comparing the N_m_/S_m_ ratios between two groups of mutations (the NSRF1 method), we now carried out stricter statistical tests that investigate the increasing or decreasing trend of N_m_/S_m_ ratios with the increased allele frequencies (the NSRF2 method), as a more robust indicator of the footprint of natural selection. Three trend tests, including two nonparametric tests [the Mann–Kendall (M&K) and Cox–Stuart (C&S) tests] and a linear regression method (LinRegress), were applied to detect the trend of N_m_/S_m_ ratios as a function of mutant allele frequencies (or mutant allele counts).

**Figure 1:**
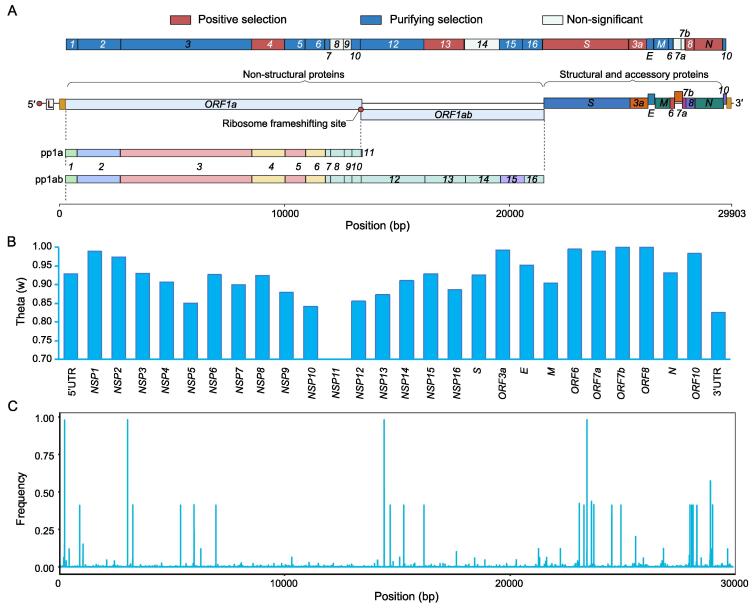
**Evidence of natural selection acting on the SARS-CoV-2 genome** **A.** Genes showing significant signals of positive selection in this study are marked in red, and those showing significant signals of negative selection in this study are in blue. The gene structures are shown at the bottom. **B.** The genetic diversity of each gene, which is indicated by Theta (w), calculated as nucleotide diversity per site in the sequences. **C.** The mutation frequency spectrum.

**Figure 2:**
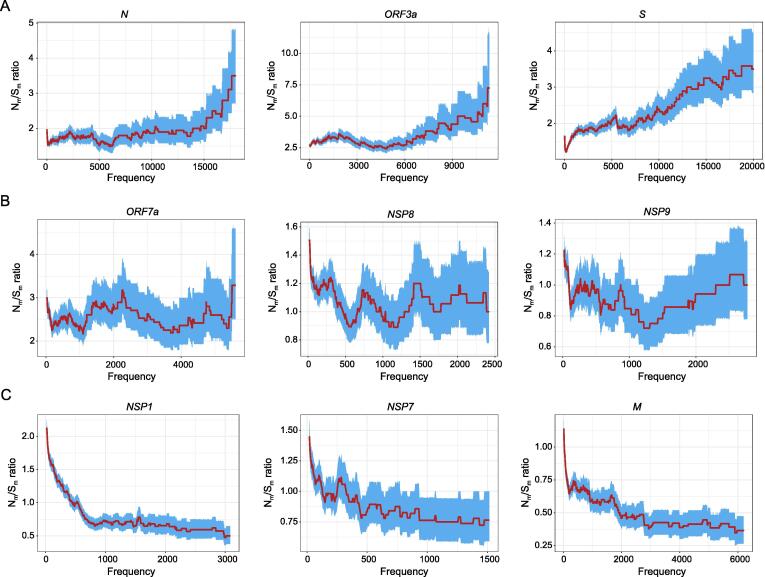
**Illustration of the trends of N_m_/S_m_ ratiosalong with the increased allele frequencies for genes with strong evidence of positive or purifying selection** **A.** Significantly increasing trends of N_m_/S_m_ ratios with the elevated allele frequencies for *N*, *ORF3a*, and *S* genes, indicative of signals of positive selection. **B.** The insignificant trends for *ORF7a*, *NSP8*, and *NSP9* genes, demonstrating no selection. **C.** Significantly decreasing trends of N_m_/S_m_ ratios with the elevated allele frequencies for *NSP1*, *NSP7*, and *M* genes, indicative of signals of purifying selection. N_m_/S_m_, the ratio of nonsynonymous *vs*. synonymous mutations.

Six genes, including spike (*S*), nucleocapsid (*N*), open reading frame 3 (*ORF3a*), *ORF8*, non-structural protein 4 (*NSP4*), and *NSP13*, show strong signals of positive selection, *i.e.*, a significantly increasing trend of N_m_/S_m_ ratios with increased allele frequencies (*P* < 0.01; Figure 1, Figure 2A and B; Table S1). Of them, *S*, *N*, *ORF3a*, and *ORF8* have been previously studied with elevated protein evolutionary rates in SARS-CoV-2 evolution [27]. These genes play critical roles in molecular processes involving pathogen–host interactions, including viral invasion into and egress from host cells, and viral inhibition and evasion of host immune response, contributing to divergent pathogenic outcomes. Intriguingly, S protein is under positive selection, indicating that it has experienced adaptive alterations in its binding affinity to human angiotensin-converting enzyme 2 (ACE2) to gain cellular entry efficiency and viral infectivity during pandemic. N protein represents one of the most crucial structural components that facilitate viral replication, assembly, and release, and acts as an important immunodominant antigen. It has been reported to promote NLRP3 inflammasome activation and induce excessive inflammatory responses [28]. N protein used to be highly conserved [29], while it is under positive selection during the SARS-CoV-2 pandemic. ORF3a has been demonstrated to induce cellular apoptosis, lysosomal exocytosis-mediated viral egress, type I IFN response inhibition, and potential cytokine storm, which belong to the key processes determining viral infectivity, pathogenicity, and virulence [30], [31]. ORF8 mediates host immune evasion through down-regulation of MHC-1 and inhibition of type I IFN response, promotes viral replication, induces apoptosis, and modulates ER stress [32]. NSP4 possibly participates in membrane rearrangement to benefit the viral replication and transcription complex formation, which may have also experienced positive selection when shifting from non-primate hosts to humans with some mutations potentially contributing to unique biological, pathological, and epidemiological features of SARS-CoV-2 [14]. NSP13 inhibits type I IFN response by interaction with TBK1, and counteracts antiviral immunity through hijack of host deubiquitinase USP13 [33]. These findings highlight that during the COVID-19 global pandemic, positive selection is very likely an essential driving force acting on viral invasion, and interplay between infection and host immune system defense, thus reshaping the viral features of infectivity, pathogenicity, and virulence.

In contrast, 14 viral genes demonstrate a significantly decreasing trend of N_m_/S_m_ ratios with increased allele frequencies (*P* < 0.01; Figure 1, Figure 2B and C; Table S1), being consistent with former section that purifying selection dominates. Ten of the negatively selected genes (*NSP1*, *NSP2*, *NSP3*, *NSP5*, *NSP6*, *NSP7*, *NSP10*, *NSP12*, *NSP15*, and *NSP16*), encode non-structural proteins of SARS-CoV-2, including components of replication and transcription complexes such as RNA-dependent RNA polymerase (RdRp), papain-like protease (PLpro), main proteinase (Mpro), and RNA primase, which are all essential to viral RNA replication, transcription, and translation [34], [35]. NSP10 and NSP16 (2′-O-methyltransferase) form a complex during coronavirus life cycle, which can methylate 5′ cap of viral RNAs, enhancing their translation and mimicking cellular mRNAs to prevent recognition by host innate immunity [36]. Other two accessory genes (*ORF6* and *ORF10*) play roles in evading host immune restriction. ORF6 has been demonstrated to inhibit type I IFN response via blocking nuclear translocation of STAT2, STAT2, and IRF3, and prevent host immune response via nuclear imprisonment of host mRNAs, serving as an antagonist of host immunity [37]. The rest negatively selected structural genes encode viral envelope (E) and membrane (M) proteins, and are involved in the assembly of progeny virions [38], [39]. These results indicate that proteins conferring coronaviral fundamental molecular functions, such as viral replication, translation, assembly, and functions in evasion of host innate and adaptive immune systems (like mimicking or imprisonment of host mRNAs), are under significant purifying selection.

### Clustering pattern of viral lineages

**Figure 3:**
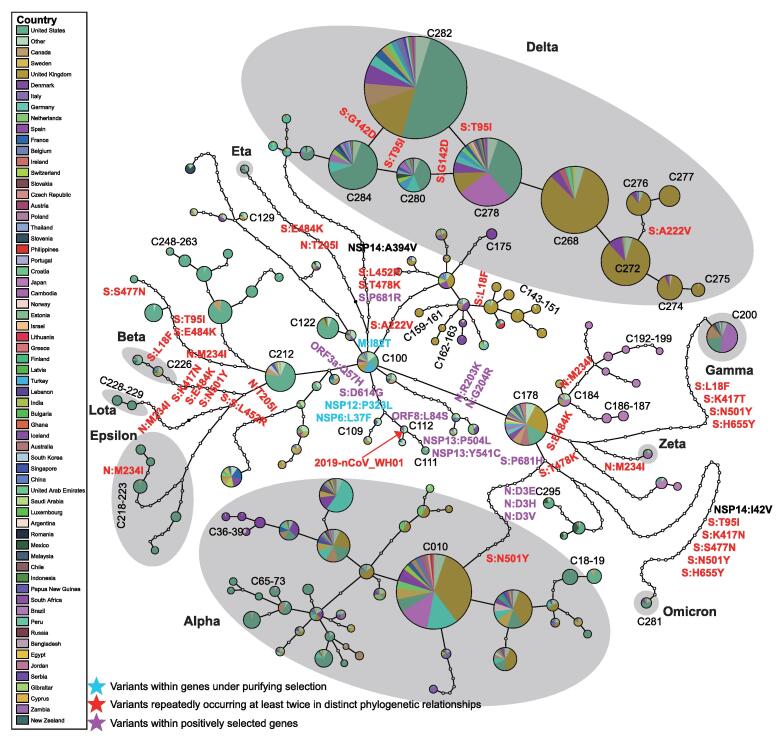
**The genealogical relationship of worldwide haplotype clusters of SARS-CoV-2** The nodes represent different haplotype clusters, with the node sizes proportional to the counts of the belonged sequences. The number of line segments separated by dots between adjacent nodes indicates the hamming distance between clusters. Within each node, its geographical distribution is presented. The listed mutations differentiating adjacent clusters are marked in purple for those within genes under positive selection, in cyan for those within genes under purifying selection, and in red for those repeatedly occurring at least twice in distinct phylogenetic relationships.

As we have demonstrated, although the viral genomes are overall under purifying selection, positive selection has been driving the evolution of genes related to coronavirus infection and host immune system defense, probably shaping epidemic and pathogenic diversification of viral populations. A further step is to understand the spatial–temporal dynamics of the diversification and identify the putative functional mutations subject to positive selection. Some studies have provided a list of candidate mutations, most of which were identified according to the trends of allele frequency changes in the overall global samples. As we know, the viral populations have been evolving and spreading in heterogeneous rates, demonstrating a clustering pattern. Investigating the allele frequencies in the pooled samples from multiple populations has two limitations: first, it has limited power to identify mutants which arose in a local population recently while are in a low frequency in the global population; second, it provides little information on the spatial and temporal origins (when and where) of these functional mutations. To track the evolutionary dynamics of genomic variants in a fine scale, we partitioned the sample of 3,328,405 genomes into distinct clusters according to their sequence similarity and evolutionary relationship, and identified 138 worldwide predominant clusters of SARS-CoV-2, denominated as C10, C23, …, and C299, respectively (see the Materials and methods section for details of the partition approach). The clusters and their genealogical relationship on a haplotype network are presented in Figure 3.

The genealogical relationship reflects establishment and evolutionary routines of diversified viral clusters, consisting of repetitive processes of viral emergence, transmission across populations and countries, and mutation accumulation as well. As shown in Figure 3, the viruses represent extensive transmissions across continents and countries along with time. The haplotype clusters comprise the currently circulating variants of concern (VOCs) and variants of interest (VOIs) like the B.1.1.7/Alpha (UK), B.1.351/Beta (South Africa), P.1/Gamma (Brazil), B.1.617/Delta (India), B.1.1.529/Omicron (South Africa), B.1.427/429/Epsilon (the State of California, USA), B.1.525/Eta (UK and Nigeria), P.2/Zeta (Brazil), and B.1.526/Lota (the State of New York, USA) lineages. Emergence of VOCs and VOIs is usually accompanied by accumulation of an excess of mutations, while VOC is characterized by signal of enhanced transmission [40]. Alpha and Delta variants are the most abundant and widely distributed haplotype clusters so far, which broadly ravage USA and Europe (especially in UK). Of note, the clusters are still subject to ongoing evolution and branching, which warrant further surveillance.

### Tracking the spatial and temporal occurrence of putatively selected mutations along the pandemic dynamics

Nucleotide mutations that are predominant in a cluster and absent or in low frequencies in others are potentially of functional importance for viral pathogenicity and transmissibility, of which some may be the targets of positive selection. Following this criterion, we identified 545 protein-coding variations differentiated among the 138 delineated haplotype clusters, including 361 N_m_ and 175 S_m_ sites. We mapped the occurrence of some of these mutations to branches connecting the clusters on the haplotype network (Figure 3). The numbers of inter-cluster mutations per branch vary from 1 to 47. We should emphasize that the allele frequency of a single mutant locus is not informative or robust to test the effect of natural selection; the list of mutations identified in this section serves as the mostly putative candidate loci under positive selection for further functional investigation. The evidence of natural selection was discussed using the trend of N_m_/S_m_ ratios in former sections.

The C112 is likely the earliest cluster if using sample collection dates as a criterion [also with the inferred Time to the Most Recent Common Ancestor (TMRCA), results not shown]. A N_m_ site L84S in the ORF8 protein, together with a tightly linked S_m_ variant (S76S) in the NSP4 protein, emerged on the branch, leading to other primary early clusters like C109 and C100 (Figure 3). The L84S replacement together with S76S were used in former studies to define two major haplotype groups in the early epidemic stage: the L and S lineages. The proportion of L lineage in samples collected before and after Wuhan lockdown showed distinct differentiation (99% *vs*. 70%), and it was hypothesized that the frequency change of L lineage may due to different pressures of negative selection from containment measures [41]. It is disputable for that the allele frequency change can be caused by sampling bias and clustering infection as well [25]. In the map of haplotype network, L84S is pinpointed to the branch connecting C112 and the ancestral node of C109 and C100 (Figure 3), with a very low frequency of 0.2627% in C112 and increased to 100% in both C109 and C100, demonstrating an obvious cluster expanding pattern in the fine scale. According to COVID-3D database [42], the L84S amino acid alteration was predicted to eliminate 4 hydrophobic bonds and lead to destabilization of ORF8 protein. Another study with computational protein modeling proposed that L84S can mitigate the binding of ORF8 to human complement C3b, which is negatively regulated by the C-terminal serine-protease catalytic domain of the human complement factor 1, and activates the host complement system [43]. Therefore, the L84S mutation possibly impacts the normal function of ORF8*,* and plays an important role in the host immune responses and infection outcome.

The N_m_ site D614G in the epitope region (the receptor binding domain) of S protein occurred in the common ancestor of C100 and majority of the clusters (Figure 3), which has been demonstrated to be associated with enhanced binding to the human ACE2, and increased viral replication, transmissibility, and loads in upper respiratory tract, indicating a competitive fitness advantage in humans [10], [23], [24], [44]. Another N_m_ site P323L in NSP12 (a kind of RdRp) also occurred at this stage, coupling the evolution with D614G. This mutation might regulate the activity of RdRp, and is related to viral replication and fidelity, altering SARS-CoV-2 mutation rates [45].

Derived from C100, two N_m_ sites, R203K and G204R, on the phosphoprotein domain of N protein arose to 100% in C178. Both clusters are widely distributed in European, Asian, and American countries. According to the structural prediction provided by the COVID-3D database [42], R203K and G204R both destabilize the N protein, and the predicted actual free energy value (ΔΔG) using the mutation Cutoff Scanning Matrix (ΔΔG^stability^ mCSM) are −1.71 kcal/mol and −1.07 kcal/mol, respectively, resulting in the alteration of their molecular interactions with other amino acids, such as carbonyl, polar bonds, and hydrogen bonds (Figure S1A–D). Mutations on N protein may be functionally relevant to viral replication and assembly, and participate in immune evasion and viral infections [39], [46], [47], [48]. Diverged from C100, the Q57H mutation in ORF3a, one of the positively selected genes, arose and fixed in the C212, C240, and C242 clusters. This variant was predicted to exert structural destabilization (ΔΔG^stability^ mCSM = −1.55 kcal/mol) (Figure S1E and F) and deleterious effects on protein function [49]. Another mutation I82T within the third membrane spanning helices in M protein diverges C102 and C293 (the early clusters of Delta variant) from C100, which is implicated in viral glucose transport. This mutation is structurally predicted to destabilize the M protein with ΔΔG^stability^ mCSM of −2.9 kcal/mol, and it is observed that it may be associated with the emergence of clusters with an excess of mutations.

Mutations in NSP14, an error-correcting exonuclease protein, may lead to malfunction of ExoN proofreading activity, thus resulting in elevated mutation rates during viral replication [40], [50]. In our case, the majority of mutations in NSP14 were associated with elevated mutation rates, especially A394V and I42V that were relevant to the emergence of Delta (C293) and Omicron (C281) variants. A394V was predicted to destabilize NSP14 with estimated ΔΔG^stability^ mCSM of −0.55 kcal/mol, and I42V with ΔΔG^stability^ mCSM of −1.06 kcal/mol. We proposed that mutations in NSP14 could be potential predictors for clusters with a high mutation rate.

### Mutations with rapid increase of frequency within clusters are potential targets of selection

Other than the mutations demonstrating nearly fixed divergence among clusters, mutations presenting prominent frequency increasing trends over sampling times within a cluster are also potential sites with evolutionary or functional significance in COVID-19 epidemic. The mutations having increasing trends in frequency independently within at least two clusters are of significance in evolution and function with high confidence. We profiled those within-cluster mutations according to their frequency dynamics during a period of 30 sequential sampling times, and 11 mutations demonstrating a significant trend of increased frequency over sampling times (simultaneously tested by M&K, C&S, and LinRegress tests; *P* < 0.005) independently within at least two clusters were identified (Figure S2).

Within C162 and C39 clusters that both predominate in Denmark, a N_m_ site L85F in the positively selected gene *ORF3a* displayed independently increased frequencies from 0 to 0.62 and from 0 to 1 in two different periods of November 09, 2020–March 01, 2021 and March 29, 2021–August 18, 2021, respectively. This variation located in the second transmembrane segment of the ORF3a protein and potentially can affect the function of virus-induced cell apoptosis and viral egress of SARS-CoV-2, as well as host immune responses and clinical outcomes.

A N_m_ site V1264L of S protein independently rises its frequencies from 0 to 0.30 within C276 and from 0 to 0.20 within C129. This variation is located in the cysteine rich intravirion region at the C-terminus of coronavirus S protein, in which the cysteine residues are targets of palmitoylation, necessary for efficiently S incorporation into virions and S-mediated membrane fusions that impact the efficiency of host cellular entry thus viral infectivity [51].

As mentioned above, mutations in NSP14, the error-correcting exonuclease protein, may be strongly associated with elevated mutation rates, and be of first priority to be monitored. We observed a substitution of M72I in NSP14 having independent increases in frequency from 0 to 0.60 within C295 and from 0 to 0.10 within Delta C129. Intriguingly, we observed consistent ongoing rises in frequency across multiple European countries including UK, Norway, Belgium, Italy, Germany, and Netherlands (Figure S2). M72I is close to the sites at the heterodimer interface of NSP14/NSP10 complex which stimulates the ExoN activity of NSP14, which may elevate the mutation rates of SARS-CoV-2. Moreover, it is predicted to structurally destabilize NSP14 or NSP14/NSP10 complex with a significant ΔΔG^stability^ mCSM of −1.89 kcal/mol. Again, the findings suggest that mutations in NSP14 are supposed to be under constant surveillance in future, and the clusters of Delta variant are still under ongoing selection, which warrants further attentions.

## Discussion

Identification of the evolutionary dynamics of SARS-CoV-2 during its pandemic in worldwide human populations, is confronted with great challenges. The dynamics of viral populations demonstrates a series of founder events caused by clustering infection or bursts of epidemic in local regions. Besides, the genomic samples are usually collected from different times and locations disproportionally (sampling bias). Both significantly impact the allele frequencies and bring challenges in analyzing the viral genomic data with most population genetic methods [25]. Comparing the relative excess of nonsynonymous and synonymous substitutions is relatively robust to population size changes, representing an efficient approach for evaluating the effects of natural selection on SARS-CoV-2. The approaches we used in this study are logically similar to the known McDonald–Kreitman test [52], [53], [54] in molecular evolution, which compares the ratio of nonsynonymous to synonymous substitutions of between-species divergence to that of within-species polymorphisms, and uses the latter as an internal control under neutrality. In contrast, the method we proposed here, referred as the NSRF1 method, is for only comparing genetic polymorphisms within a species. The method is novel in using the N_m_/S_m_ ratio of mutations with very low frequencies as the internal control under neutral evolution. The fact that the population dynamics of mutants with very low frequencies is identical to those under neutrality is valid according to diffusion theory for the allele frequency in a large population [26]. When identifying natural selection acting on individual genes, we further investigate the increasing or decreasing trend of N_m_/S_m_ ratios along with the increased mutant allele frequencies under the assumption that the mutations with higher frequencies tend to undergo a longer duration of natural selection and present proportionally increasing or decreasing N_m_/S_m_ ratios (the NSRF2 method), which are more efficient and robust indicators of natural selection.

By adopting the strategies, we demonstrate with multiple lines of evidence that SARS-CoV-2 genomes are overall constrained under purifying selection during its pandemic. In spite of this, evidence of positive selection acting on specific genes that participate in coronavirus infection and host immune evasion are intriguingly observed. These results indicate that ongoing positive selection is actively driving tighter affinity with human and escape of host antiviral immunity, leading to high transmissibility and mild symptom in a long-run evolution of SARS-CoV-2. Such trend was supported by studies analyzing the immunological and epidemiological data on endemic human coronaviruses [55].

We further partition the viral genomic samples into 138 haplotype clusters according to their sequence similarity and genealogical relationship. Superimposing on the 138 worldwide transmission clusters, we provide a list of 556 mutations as putative target sites of natural selection. Whilst there is no concrete evidence supporting their functional significance during the outbreaks, mutations showing between-cluster divergence or within-cluster frequency boost may explain distinct pathogenicity and infectivity. Thus, the list of mutations provides a basis for further functional study and clinical treatment.

## Materials and methods

### SARS-CoV-2 genomes downloaded from public databases

SARS-CoV-2 genomic sequences were downloaded from the 2019 Novel Coronavirus Resource (RCoV19, https://bigd.big.ac.cn/ncov/) [56] and the Global Initiative on Sharing All Influenza Data (GISAID, https://www.gisaid.org/). A total of 3,328,405 sequences from 169 countries were included, with the sampling dates ranging from December 24, 2019 to December 30, 2021.

### Identification of nucleotide mutations

All the sequences were aligned using MUSCLE [57] with default parameter settings. Then, 265 bp of the 5′-untranslated region (UTR) and 229 bp of the 3′-UTR region were trimmed out, with a final length of 29,409 nucleotides retained. Nucleotide mutations were called by comparing these sequences with the reference sequence (NC_045512).

### Identification of selection on genomes or individual genes

Selection on viral genomes was detected by comparing the relative abundance of N_m_ and S_m_ between mutations with high and low allele frequencies (referred to as the NSRF1 method), between widespread and non-widespread mutations, and between mutations with long- and short-time spanning. Selection on individual genes was identified as follows. We divided mutations into 4000 bins corresponding to ≥ 5, ≥ 10, …, and ≥ 20,000 derived allele counts. Let $r_{j}$ denote the N_m_/S_m_ ratio for mutations with the derived allele counts ≥j. When under purifying selection, $r_{j}$ values are expected to decrease with j; while under positive selection, an increasing trend of $r_{j}$ values is expected. Thus, we applied three kinds of statistical methods to detect the increasing or decreasing trends of $r_{j}$ values as a function of *j*, including M&K and C&S tests, and LinRegress (referred to as the NSRF2 method). The false discovery rate correction was performed to correct for false positives.

### Clustering definition of viral lineages based on the haplotype network analysis

Each viral haplotype was assigned to a cluster following the steps of the classification tree shown in Figure S3. A total of 545 SNPs were chosen as the features for the classification. Each of the haplotypes was assigned to different clusters according to their alleles on the 545 SNP loci following the order of the features.

### Prediction of the effects of mutations on protein function

An online resource COVID-3D (http://biosig.unimelb.edu.au/covid3d/) was used to predict the effects of mutations of SARS-CoV-2 on protein structure [42].

## CRediT author statement

**Yali Hou:** Formal analysis, Methodology, Writing - original draft, Writing - review & editing. **Shilei Zhao:** Methodology, Formal analysis, Visualization. **Qi Liu:** Formal analysis, Methodology, Visualization. **Xiaolong Zhang:** Formal analysis, Visualization. **Tong Sha:** Formal analysis. **Yankai Su:** Formal analysis. **Wenming Zhao:** Resources. **Yiming Bao:** Resources. **Yongbiao Xue:** Conceptualization, Writing - original draft, Supervision. **Hua Chen:** Conceptualization, Methodology, Writing - original draft, Writing - review & editing, Supervision. All authors have read and approved the final manuscript.

## Competing interests

The authors have declared no competing interests.

## Supplementary material

Supplementary Figure S1 **The impact of mutations on protein structures A.** and **C**. The predicted 3D structure for N protein. **B**. The alteration of molecular interactions by introducing the R203K mutation. **D**. The alteration of molecular interactions by introducing the G204R mutation. **E**. The predicted 3D structure for ORF3a protein. **F**. The alteration of molecular interactions by introducing the Q57H mutation. N, nucleocapsid; VDW, Van der Waals interaction.

Supplementary Figure S2 **The 11 mutations presenting prominent frequency increasing trend over sampling times (simultaneously tested by M&K, C&S, and LinRegress tests, *P* < 0.005) independently within at least 2 clusters, as potential targets of selection**

Supplementary Figure S3 **The schematic diagram of classification tree defining the viral haplotype clusters** The viral genomes were assigned to different clusters according to the alleles of 545 featured SNPs. The classification tree is bifurcated according to distinct alleles of a featured SNP with its position marked in the node oval, and the alleles are listed on the branches. We denominated each node starting from the top in a numerical order as C1, C2, C3, … . Ultimately, the leaf nodes (rectangle) with the denomination are defined as haplotype clusters. SNPs, single nucleotide polymorphisms.

Supplementary Table S1 **Three trend tests to detect natural selection acting on individual genes**

## References

[1] Gorbalenya A.E., Baker S.C., Baric R.S., de Groot R.J., Drosten C., Gulyaeva A.A. (2020). The species *Severe acute respiratory syndrome-related coronavirus*: classifying 2019-nCoV and naming it SARS-CoV-2. Nat Microbiol.

[2] Wu F., Zhao S., Yu B., Chen Y.M., Wang W., Song Z.G. (2020). A new coronavirus associated with human respiratory disease in China. Nature.

[3] Zhou P., Yang X.L., Wang X.G., Hu B., Zhang L., Zhang W. (2020). A pneumonia outbreak associated with a new coronavirus of probable bat origin. Nature.

[4] Zhu N., Zhang D., Wang W., Li X., Yang B., Song J. (2020). A novel coronavirus from patients with pneumonia in China, 2019. N Engl J Med.

[5] Domingo E., Sheldon J., Perales C. (2012). Viral quasispecies evolution. Microbiol Mol Biol Rev.

[6] Day T., Gandon S., Lion S., Otto S.P. (2020). On the evolutionary epidemiology of SARS-CoV-2. Curr Biol.

[7] Geoghegan J.L., Holmes E.C. (2018). The phylogenomics of evolving virus virulence. Nat Rev Genet.

[8] Lv M., Luo X., Estill J., Liu Y., Ren M., Wang J. (2020). Coronavirus disease (COVID-19): a scoping review. Euro Surveill.

[9] Zhao S., Chen H. (2020). Modeling the epidemic dynamics and control of COVID-19 outbreak in China. Quant Biol.

[10] Korber B., Fischer W.M., Gnanakaran S., Yoon H., Theiler J., Abfalterer W. (2020). Tracking changes in SARS-CoV-2 Spike: evidence that D614G increases infectivity of the COVID-19 virus. Cell.

[11] Dorp C.H.V., Goldberg E.E., Hengartner N., Ke R., Romero-Severson E.O. (2021). Estimating the strength of selection for new SARS-CoV-2 variants. Nat Commun.

[12] Shuai H., Chan J.F.W., Hu B., Chai Y., Yuen T.T.T., Yin F. (2022). Attenuated replication and pathogenicity of SARS-CoV-2 B.1.1.529 Omicron. Nature.

[13] Andersen K.G., Rambaut A., Lipkin W.I., Holmes E.C., Garry R.F. (2020). The proximal origin of SARS-CoV-2. Nat Med.

[14] Berrio A., Gartner V., Wray G.A. (2020). Positive selection within the genomes of SARS-CoV-2 and other Coronaviruses independent of impact on protein function. PeerJ.

[15] Cagliani R., Forni D., Clerici M., Sironi M. (2020). Computational inference of selection underlying the evolution of the novel coronavirus, severe acute respiratory syndrome coronavirus 2. J Virol.

[16] Chaw S.M., Tai J.H., Chen S.L., Hsieh C.H., Chang S.Y., Yeh S.H. (2020). The origin and underlying driving forces of the SARS-CoV-2 outbreak. J Biomed Sci.

[17] Jungreis I., Sealfon R., Kellis M. (2021). SARS-CoV-2 gene content and COVID-19 mutation impact by comparing 44 *Sarbecovirus* genomes. Nat Commun.

[18] Li X., Giorgi E.E., Marichannegowda M.H., Foley B., Xiao C., Kong X.P. (2020). Emergence of SARS-CoV-2 through recombination and strong purifying selection. Sci Adv.

[19] Kumar S., Tao Q., Weaver S., Sanderford M., Caraballo-Ortiz M.A., Sharma S. (2021). An evolutionary portrait of the progenitor SARS-CoV-2 and its dominant offshoots in COVID-19 pandemic. Mol Biol Evol.

[20] Rochman N.D., Wolf Y.I., Faure G., Mutz P., Zhang F., Koonin E.V. (2021). Ongoing global and regional adaptive evolution of SARS-CoV-2. Proc Natl Acad Sci U S A.

[21] Velazquez-Salinas L., Zarate S., Eberl S., Gladue D.P., Novella I., Borca M.V. (2020). Positive selection of *ORF1ab*, *ORF3a*, and *ORF8* genes drives the early evolutionary trends of SARS-CoV-2 during the 2020 COVID-19 pandemic. Front Microbiol.

[22] Hu J., He C.L., Gao Q.Z., Zhang G.J., Cao X.X., Long Q.X. (2020). D614G mutation of SARS-CoV-2 spike protein enhances viral infectivity. bioRxiv.

[23] Volz E., Hill V., McCrone J.T., Price A., Jorgensen D., O'Toole A. (2021). Evaluating the effects of SARS-CoV-2 spike mutation D614G on transmissibility and pathogenicity. Cell.

[24] Zhou B., Thao T.T.N., Hoffmann D., Taddeo A., Ebert N., Labroussaa F. (2021). SARS-CoV-2 spike D614G change enhances replication and transmission. Nature.

[25] Liu Q., Zhao S., Shi C.M., Song S., Zhu S., Su Y. (2020). Population genetics of SARS-CoV-2: disentangling effects of sampling bias and infection clusters. Genomics Proteomics Bioinformatics.

[26] Kimura M. (1979). The neutral theory of molecular evolution. Sci Am.

[27] Wei C., Chen Y.M., Chen Y., Qian W. (2021). The missing expression level-evolutionary rate anticorrelation in viruses does not support protein function as a main constraint on sequence evolution. Genome Biol Evol.

[28] Pan P., Shen M., Yu Z., Ge W., Chen K., Tian M. (2021). SARS-CoV-2 N protein promotes NLRP3 inflammasome activation to induce hyperinflammation. Nat Commun.

[29] Casasanta M.A., Jonaid G.M., Kaylor L., Luqiu W.Y., Solares M.J., Schroen M.L. (2021). Microchip-based structure determination of low-molecular weight proteins using cryo-electron microscopy. Nanoscale.

[30] Chen D., Zheng Q., Sun L., Ji M., Li Y., Deng H. (2021). ORF3a of SARS-CoV-2 promotes lysosomal exocytosis-mediated viral egress. Dev Cell.

[31] Ren Y., Shu T., Wu D., Mu J., Wang C., Huang M. (2020). The ORF3a protein of SARS-CoV-2 induces apoptosis in cells. Cell Mol Immunol.

[32] Flower T.G., Buffalo C.Z., Hooy R.M., Allaire M., Ren X., Hurley J.H. (2021). Structure of SARS-CoV-2 ORF8, a rapidly evolving immune evasion protein. Proc Natl Acad Sci U S A.

[33] Guo G., Gao M., Gao X., Zhu B., Huang J., Luo K. (2021). SARS-CoV-2 non-structural protein 13 (nsp13) hijacks host deubiquitinase USP13 and counteracts host antiviral immune response. Signal Transduct Target Ther.

[34] Wang R., Hozumi Y., Yin C., Wei G.W. (2020). Decoding SARS-CoV-2 transmission and evolution and ramifications for COVID-19 diagnosis, vaccine, and medicine. J Chem Inf Model.

[35] Yoshimoto F.K. (2020). The proteins of severe acute respiratory syndrome coronavirus-2 (SARS CoV-2 or n-COV19), the cause of COVID-19. Protein J.

[36] Viswanathan T., Arya S., Chan S.H., Qi S., Dai N., Misra A. (2020). Structural basis of RNA cap modification by SARS-CoV-2. Nat Commun.

[37] Miorin L., Kehrer T., Sanchez-Aparicio M.T., Zhang K., Cohen P., Patel R.S. (2020). SARS-CoV-2 Orf6 hijacks Nup98 to block STAT nuclear import and antagonize interferon signaling. Proc Natl Acad Sci U S A.

[38] Kim D., Lee J.Y., Yang J.S., Kim J.W., Kim V.N., Chang H. (2020). The architecture of SARS-CoV-2 transcriptome. Cell.

[39] Astuti I., Ysrafil. (2020). Severe Acute Respiratory Syndrome Coronavirus 2 (SARS-CoV-2): an overview of viral structure and host response. Diabetes Metab Syndr.

[40] Otto S.P., Day T., Arino J., Colijn C., Dushoff J., Li M. (2021). The origins and potential future of SARS-CoV-2 variants of concern in the evolving COVID-19 pandemic. Curr Biol.

[41] Tang X., Wu C., Li X., Song Y., Yao X., Wu X. (2020). On the origin and continuing evolution of SARS-CoV-2. Natl Sci Rev.

[42] Portelli S., Olshansky M., Rodrigues C.H.M., D’Souza E.N., Myung Y., Silk M. (2020). COVID-3D: an online resource to explore the structural distribution of genetic variation in SARS-CoV-2 and its implication on therapeutic development. bioRxiv.

[43] Singh J., Kar S., Hasnain S.E., Ganguly S. (2020). SARS-CoV-2 ORF8 can fold into human factor 1 catalytic domain binding site on complement C3b: predict functional mimicry. bioRxiv.

[44] Plante J.A., Liu Y., Liu J., Xia H., Johnson B.A., Lokugamage K.G. (2021). Spike mutation D614G alters SARS-CoV-2 fitness. Nature.

[45] Pachetti M., Marini B., Benedetti F., Giudici F., Mauro E., Storici P. (2020). Emerging SARS-CoV-2 mutation hot spots include a novel RNA-dependent-RNA polymerase variant. J Transl Med.

[46] Ding S.W., Han Q., Wang J., Li W.X. (2018). Antiviral RNA interference in mammals. Curr Opin Immunol.

[47] Mu J., Xu J., Zhang L., Shu T., Wu D., Huang M. (2020). SARS-CoV-2-encoded nucleocapsid protein acts as a viral suppressor of RNA interference in cells. Sci China Life Sci.

[48] Zeng W., Liu G., Ma H., Zhao D., Yang Y., Liu M. (2020). Biochemical characterization of SARS-CoV-2 nucleocapsid protein. Biochem Biophys Res Commun.

[49] Issa E., Merhi G., Panossian B., Salloum T., Tokajian S. (2020). SARS-CoV-2 and ORF3a: nonsynonymous mutations, functional domains, and viral pathogenesis. mSystems.

[50] Takada K., Ueda M.T., Shichinohe S., Kida Y., Ono C., Matsuura Y. (2020). Genomic diversity of SARS-CoV-2 can be accelerated by mutations in the *nsp14* gene. bioRxiv.

[51] Shulla A., Gallagher T. (2009). Role of spike protein endodomains in regulating coronavirus entry. J Biol Chem.

[52] Bhatt S., Katzourakis A., Pybus O.G. (2010). Detecting natural selection in RNA virus populations using sequence summary statistics. Infect Genet Evol.

[53] Charlesworth J., Eyre-Walker A. (2008). The McDonald–Kreitman test and slightly deleterious mutations. Mol Biol Evol.

[54] Welch J.J. (2006). Estimating the genomewide rate of adaptive protein evolution in *Drosophila*. Genetics.

[55] Lavine J.S., Bjornstad O.N., Antia R. (2021). Immunological characteristics govern the transition of COVID-19 to endemicity. Science.

[56] Zhao W.M., Song S.H., Chen M.L., Zou D., Ma L.N., Ma Y.K. (2020). The 2019 novel coronavirus resource. Hereditas (Beijing).

[57] Edgar R.C. (2004). MUSCLE: multiple sequence alignment with high accuracy and high throughput. Nucleic Acids Res.

