# Supplementary material for: Ongoing Positive Selection Drives the Evolution of SARS-CoV-2 Genomes
Source: Genomics Proteomics Bioinformatics. 2022 Jun 26;20(6):1214–23. doi: 10.1016/j.gpb.2022.05.009 (PMC9233880; doi:10.1016/j.gpb.2022.05.009)
Supplement: Supplementary Figure S1 — The impact of mutations on protein structures A. and C. The predicted 3D structure for N protein. B. The alteration of molecular interactions by introducing the R203K mutation. D. The alteration of molecular interactions by introducing the G204R mutation. E. The predicted 3D structure for ORF3a protein. F. The alteration of molecular interactions by introducing the Q57H mutation. N, nucleocapsid; VDW, Van der Waals interaction. [file mmc1.pptx]

## Slide 1
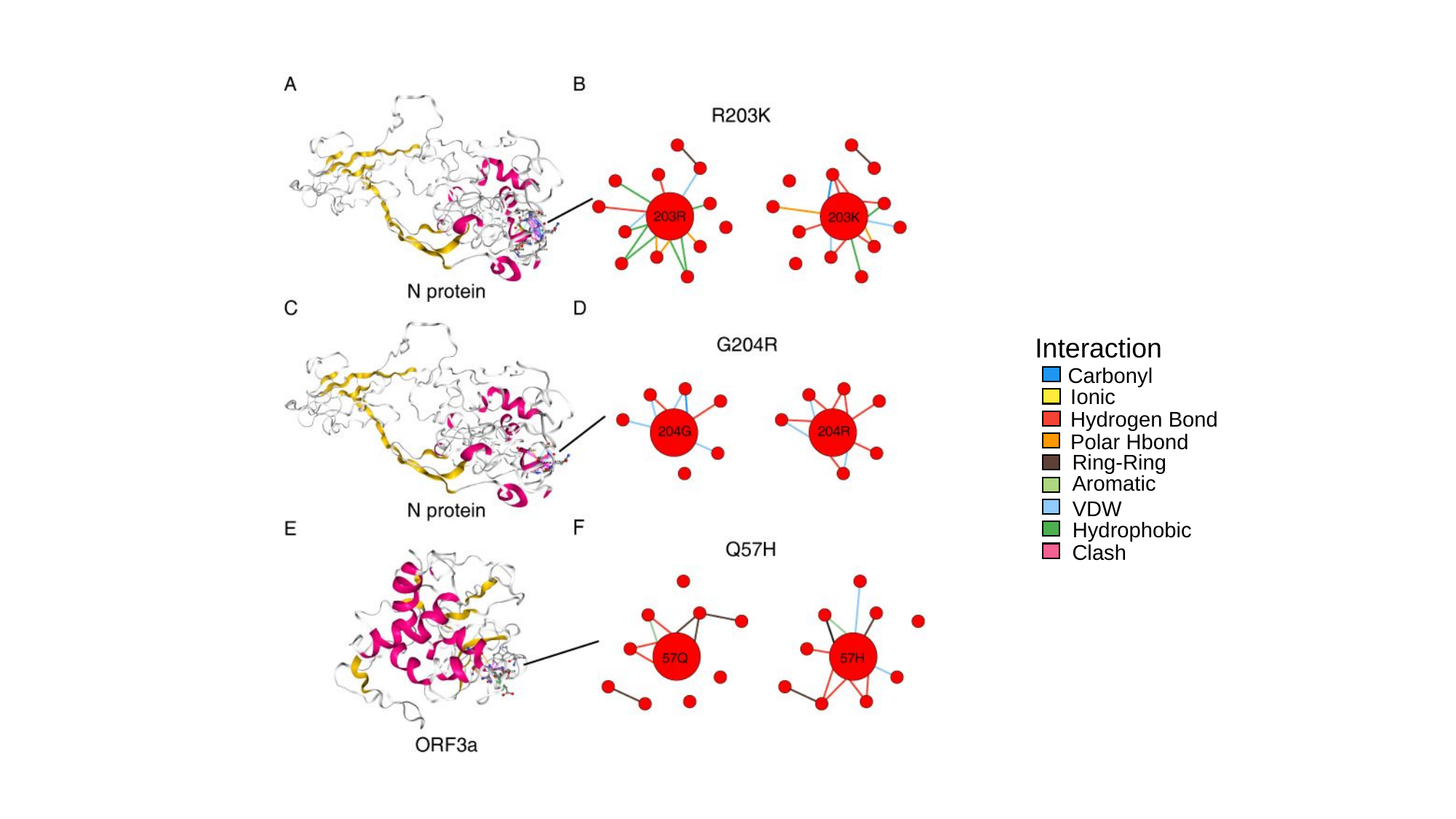

Interaction
Carbonyl
Ionic
Hydrogen Bond
Polar Hbond
Ring-Ring
Aromatic
VDW
Hydrophobic
Clash
