# Supplementary figures and images for: Ongoing Positive Selection Drives the Evolution of SARS-CoV-2 Genomes
Source: Genomics Proteomics Bioinformatics. 2022 Jun 26;20(6):1214–23. doi: 10.1016/j.gpb.2022.05.009 (PMC9233880; doi:10.1016/j.gpb.2022.05.009)

## Slide 1
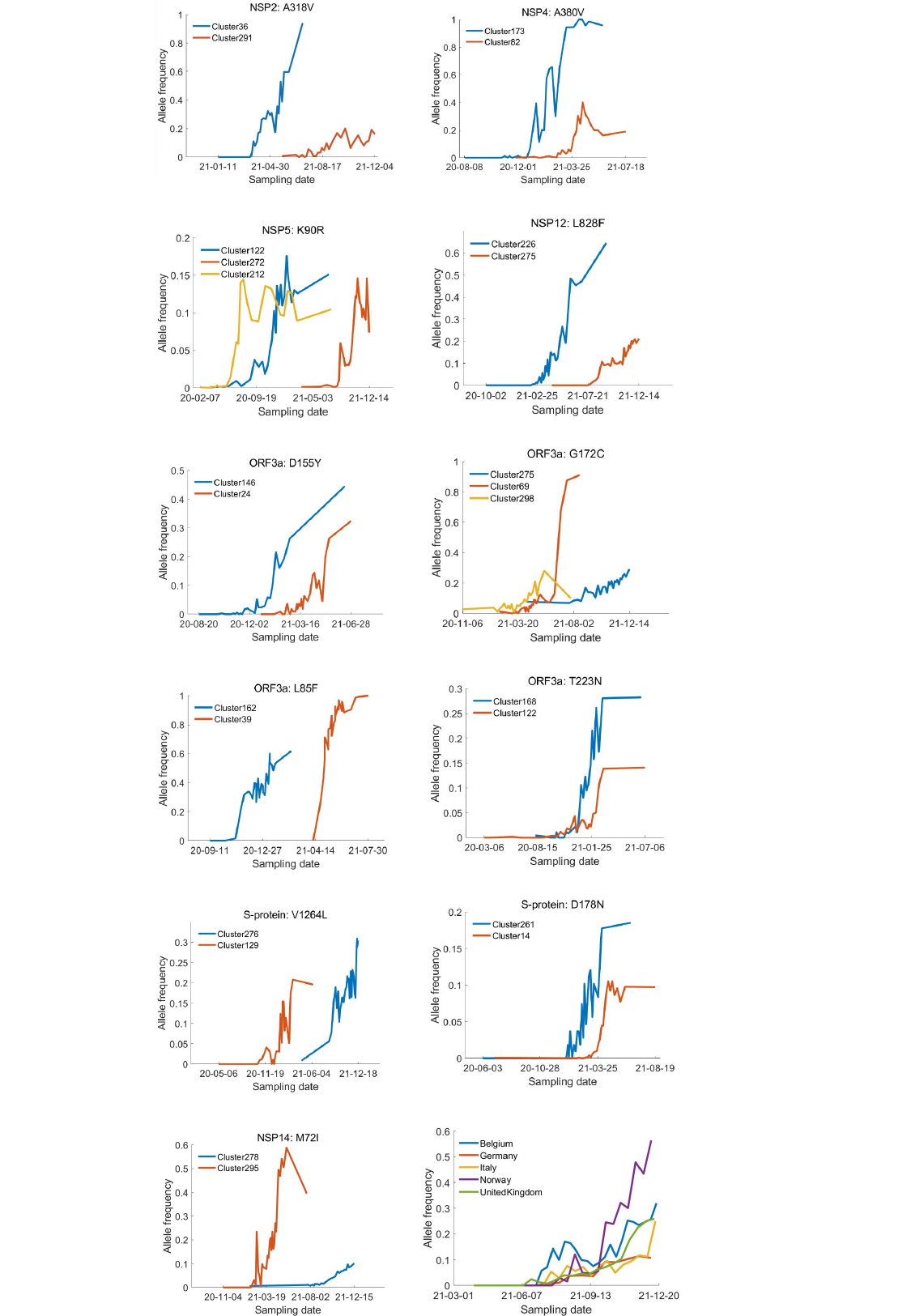

Supplement: Supplementary Figure S2 — The 11 mutations presenting prominent frequency increasing trend over sampling times (simultaneously tested by M&K, C&S, and LinRegress tests, P < 0.005) independently within at least 2 clusters, as potential targets of selection [file mmc2.pptx]

## Slide 1
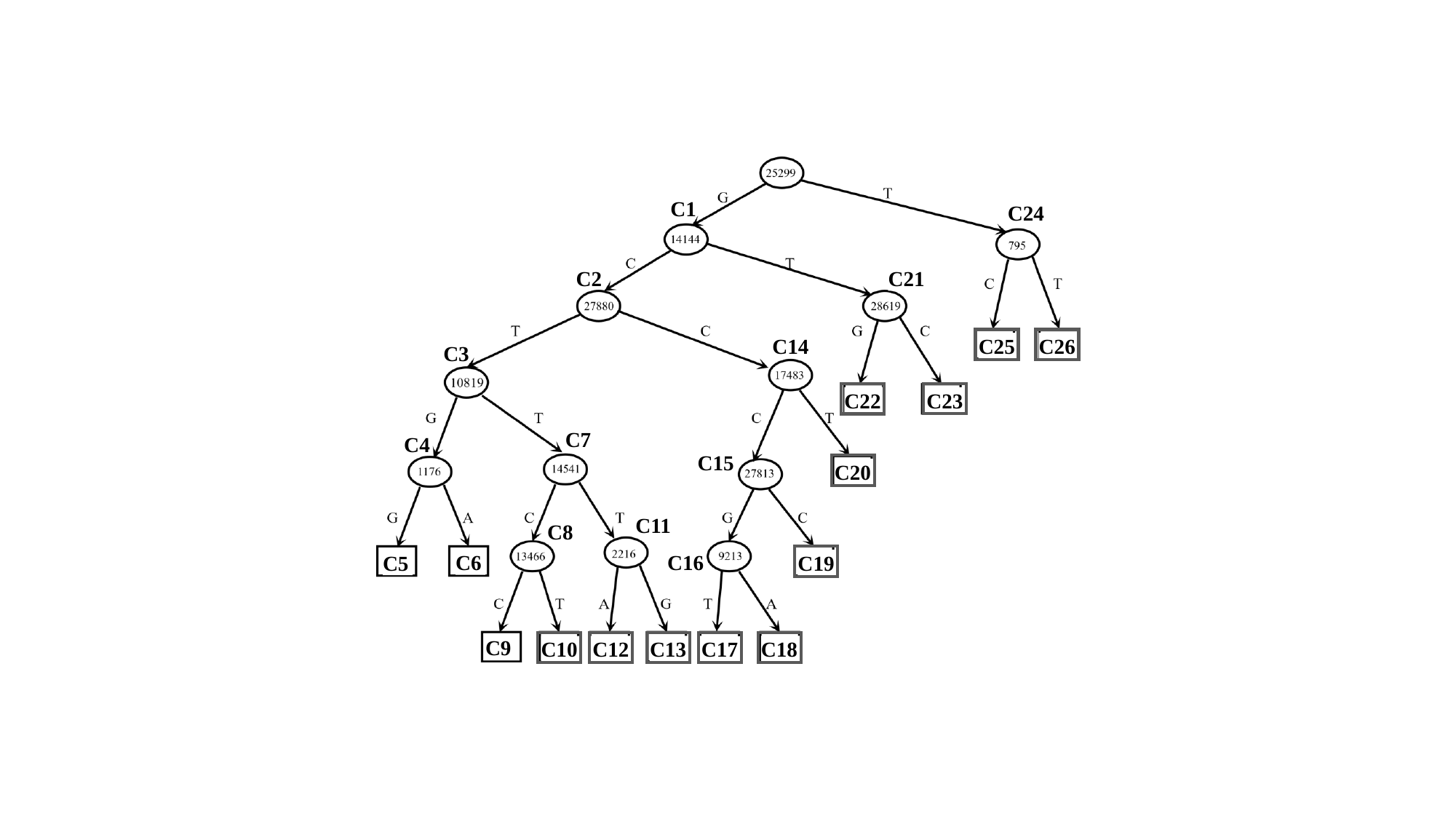

C1
C2
C21
C25
C26
C14
C3
C23
C22
C4
C7
C15
C20
C11
C8
C19
C6
C16
C5
C18
C12
C17
C9
C13
C10
C24

Supplement: Supplementary Figure S3 — The schematic diagram of classification tree defining the viral haplotype clusters The viral genomes were assigned to different clusters according to the alleles of 545 featured SNPs. The classification tree is bifurcated according to distinct alleles of a featured SNP with its position marked in the node oval, and the alleles are listed on the branches. We denominated each node starting from the top in a numerical order as C1, C2, C3, … . Ultimately, the leaf nodes (rectangle) with the denomination are defined as haplotype clusters. SNPs, single nucleotide polymorphisms. [file mmc3.pptx]
